# All-at-once multigrid approaches for one-dimensional space-fractional diffusion equations

**DOI:** 10.1007/s10092-021-00436-3

**Published:** 2021-10-07

**Authors:** Marco Donatelli, Rolf Krause, Mariarosa Mazza, Ken Trotti

**Affiliations:** 1 University of Insubria, via Valleggio 11, 22100 Como, Italy; 2 Università della Svizzera Italiana, via Giuseppe Buffi 13, 6900 Lugano, Switzerland

**Keywords:** Fractional diffusion equations, Toeplitz matrices, Spectral distribution, All-at-once systems, Space–time multigrid, 35R11, 15B05, 65F15, 68W10, 65N55

## Abstract

We focus on a time-dependent one-dimensional space-fractional diffusion equation with constant diffusion coefficients. An all-at-once rephrasing of the discretized problem, obtained by considering the time as an additional dimension, yields a large block linear system and paves the way for parallelization. In particular, in case of uniform space–time meshes, the coefficient matrix shows a two-level Toeplitz structure, and such structure can be leveraged to build ad-hoc iterative solvers that aim at ensuring an overall computational cost independent of time. In this direction, we study the behavior of certain multigrid strategies with both semi- and full-coarsening that properly take into account the sources of anisotropy of the problem caused by the grid choice and the diffusion coefficients. The performances of the aforementioned multigrid methods reveal sensitive to the choice of the time discretization scheme. Many tests show that Crank–Nicolson prevents the multigrid to yield good convergence results, while second-order backward-difference scheme is shown to be unconditionally stable and that it allows good convergence under certain conditions on the grid and the diffusion coefficients. The effectiveness of our proposal is numerically confirmed in the case of variable coefficients too and a two-dimensional example is given.

## Introduction

Fractional diffusion equations (FDEs) generalize classical partial differential equations (PDEs), and their recent success is due to the non-local behavior of fractional operators that translates in an appropriate modeling of anomalous diffusion phenomena appearing in several applicative fields, like imaging or electrophysiology [2, 4]. The non-locality of the fractional operators causes absence of sparsity in the discretization matrices. Fortunately, in presence of uniform grids, the discretization matrices show a *Toeplitz-like structure* and this paves the way for the design of iterative solvers specialized for structured linear systems.

In this regard, for one-dimensional space-FDE problems we mention the circulant preconditioning in [14], the multigrid method in [20], and the structure preserving tridiagonal preconditioners in [6]. The latter preconditioners were motivated by the spectral study of the coefficient matrices through the notion of symbol and by the so-called generalized locally Toeplitz theory [9].

In the two-dimensional setting, classical preconditioners based on multilevel circulant matrices are not well suited, while multigrid methods, possibly used as preconditioners, can be effective and robust solvers. Some multigrid proposals for FDEs discretized with finite differences are given in [16–18]. When finite element or finite volume discretizations are adopted, multigrid methods are investigated in [13] and [7], respectively. In particular, in [18] the spectral approach presented in [6] has been extended to two-dimensional FDEs and has been used to define a multigrid preconditioner which is particularly effective when the fractional orders are both close to 2. For fractional derivative orders that differ from each other, i.e., for FDEs that show an intrinsic anisotropy along the axes, we mention the “multigrid as smoother” (MG-S) approach firstly proposed in [19] for integer-order problems and then adapted to fractional ones in [5]. Such method consists in a V-cycle with semi-coarsening used as smoother inside an outer full-coarsening, and in [5] it is shown to be a robust preconditioner in presence of strong anisotropies caused by the fractional orders.

Due to the sequentiality of time integration, with none of the aforementioned approaches we can aspire towards complete independence of time of the overall computational cost. By contrary, an all-at-once rephrasing of the discretized problem over a uniform space–time grid, obtained by considering the time as an additional dimension, yields large (multilevel) Toeplitz linear systems and opens to parallelization.

In this regard, we mention the banded Toeplitz preconditioner proposed in [27] for solving non-linear space-FDEs, and the block structured preconditioner given in [3] for dealing with arbitrary dimensional space problems. In [10, 21] a Strang-type preconditioner for solving FDEs by boundary value methods has been proposed. In the case of equal left and right diffusion coefficients, we also mention the multigrid reduction in time (MGRIT) discussed in [26]. Therein, the authors consider finite elements in space and Crank–Nicolson in time, since the MGRIT is specifically tailored for one step methods.

The present paper fits within the latter framework. Precisely, our scope is to build a fast and efficient parallel-in-time structure-based multigrid solver. We fix our attention on a weighted and shifted Grünwald difference (WSGD) discretization of a one-dimensional time-dependent space-FDE with constant diffusion coefficients. We stress that this one-dimensional problem turns out to be already a tough one, due to the block structure of the coefficient matrix and to its possibly anisotropic character because of the grid choice and the diffusion coefficients.

As for the time discretization, we opt either for Crank–Nicolson (CN) or second-order backward-difference formula (BDF2) schemes. The unconditional stability of CN-WSGD has already been proven in [23]. Concerning BDF2, in [15] it was combined with a central finite difference scheme for solving space-FDEs with diffusion coefficients equal to 1. In that same paper, a proof of unconditional stability of the resulting method was given. We extend this result to the case where the space scheme is WSGD and the diffusion coefficients are not necessarily equal to each other.

Aiming at building a parallel-in-time multigrid, we consider block Jacobi as smoother, since it is parallelizable. Moreover, exploiting the Toeplitz structure of the coefficient matrices and the related symbols we define the projectors according to what has been done in the integer-order literature for both isotropic [1], and anisotropic Toeplitz linear systems [8].

The performances of the proposed multigrid strategies reveal sensitive to the choice of the time discretization scheme. Indeed, many tests, including a two-dimensional example, show that Crank–Nicolson prevents the multigrid to yield good convergence results, while BDF2 scheme allows good convergence under certain conditions on the grid and the diffusion coefficients.

The paper is organized as follows. In Sect. 2, we introduce the problem setting and we describe both CN-WGSD and BDF2-WSGD approximation methods, by giving the formal expression and the structure of the resulting matrices. In Sect. 3, we prove that BDF2-WSGD scheme is unconditionally stable. In Sect. 4, we perform an all-at-once rephrasing of the original matrices and give some results on their spectra, which are leveraged in Sect. 5 for the design of proper multigrid strategies. Several numerical experiments, also in the case of variable diffusion coefficients, are reported in Sect. 6 for testing the performances of our proposals. Finally, in Sect. 7 we draw conclusions.

## Preliminaries

In this section, we first introduce the FDE problem we are interested in (Sect. 2.1). Second, we recall the definition of multilevel Toeplitz matrix (Sect. 2.2). The latter will be needed in Sect. 4 where we perform on a all-at-once rephrasing of the given problem and we study the related spectral properties. Finally, we briefly review the combination of the chosen finite difference space-discretization with two different time discretization schemes (Sect. 2.3).

### Problem setting: a one-dimensional space-FDE

In this work, we focus on the following one-dimensional initial-boundary value space-FDE problem

1 $$\begin{aligned} \left\{ \begin{aligned} \frac{\partial u(x,t)}{\partial t}=&\ d_+ \frac{\partial ^{\alpha }u(x,t)}{\partial _{+}x^\alpha }+d_-\frac{\partial ^{\alpha }u(x,t)}{\partial _{-}x^\alpha }+v(x,t),\\&\ \ \qquad \qquad \qquad \qquad \qquad \qquad \ \ \ (x,t)\in \varOmega \times [0,T],\\ u(x,t)=&0, \qquad \qquad \qquad \qquad \qquad \qquad \ (x,t)\in \left( \mathbb {R}\setminus \varOmega \right) \times [0,T],\\ u(x,0)=&u_0(x), \quad \qquad \qquad \qquad \qquad \quad \ \ x\in \overline{\varOmega }, \end{aligned} \right. \end{aligned}$$

where $\varOmega =(a,b)$ is the space domain, $d_\pm >0$ are the diffusion coefficients, *v*(*x*, *t*) is the forcing term, and $\frac{\partial ^{\alpha }u(x,t)}{\partial _{\pm }x^\alpha }$ are the left $(+)$ and right $(-)$ fractional derivative operators of order $\alpha \in (1,2)$ with respect to variable *x* defined as

$$\begin{aligned} \frac{\partial ^\alpha u(x,t)}{\partial _{+} x^\alpha }&= \frac{1}{\Gamma (2-\alpha )}\frac{\partial ^2}{\partial x^2}\int _L^x\frac{u(\xi ,t)}{(x-\xi )^{\alpha -1}}d\xi ,\\ \frac{\partial ^\alpha u(x,t)}{\partial _{-} x^\alpha }&=\frac{1}{\Gamma (2-\alpha )}\frac{\partial ^2}{\partial x^2}\int _x^R\frac{u(\xi ,t)}{(\xi -x)^{\alpha -1}}d\xi . \end{aligned}$$

By considering the time like an additional dimension, the discretization of equation (1) yields a two-level Toeplitz matrix. In the next subsection we clarify such structure.

### Multilevel Toeplitz matrices and their symbol

Here we report the formal definition of a multilevel Toeplitz matrix.

#### Definition 1

Let $f\in \mathrm {L}^1(\left[ -\pi ,\pi \right] ^d)$ and let ${\lbrace f_{\varvec{k}}}\rbrace _{\varvec{k}\in \mathbb {Z}^d}$ be the sequence of its Fourier coefficients defined as

$$\begin{aligned} f_{\varvec{k}}:=\frac{1}{\left( 2\pi \right) ^d}\int _{[-\pi ,\pi ]^d}f(\theta )\mathrm {e}^{-\mathrm {i}\langle \varvec{k},\theta \rangle }\mathrm {d}\theta , \end{aligned}$$

where $\langle \varvec{k},\theta \rangle =\sum _{t=1}^d k_t\theta _t$ and $\text {i}^2=-1$. Then the *d*-*level Toeplitz matrix* of partial orders $\varvec{n}=\left( n_1,\ldots ,n_d\right)$ associated with *f* is

$$\begin{aligned} \mathrm {T}_N^{(d)}:=\left[ f_{\varvec{i}-\varvec{j}}\right] _{\varvec{i},\varvec{j}=\varvec{1}}^{\varvec{n}}=\left[ \cdots \left[ \left[ f_{i_1-j_1,\ldots ,i_{d}-j_{d}}\right] _{i_d,j_d=1}^{n_d}\right] _{i_{d-1},j_{d-1}}^{n_{d-1}}\cdots \right] _{i_1,j_1=1}^{n_1}, \end{aligned}$$

where $N=\prod _{i=1}^dn_i$ is the order of the matrix. The function *f* is called the *symbol* of the matrix-sequence $\lbrace \mathrm {T}_N^{(d)}(f)\rbrace _N$.

To clarify the notation, a 2-level Toeplitz matrix of order *N* generated by *f* is given by

$$\begin{aligned} \mathrm {T}_N^{(2)}(f)=\left[ \left[ f_{[i_1-j_1,i_2-j_2]}\right] _{i_2,j_2=1}^{n_2}\right] _{i_1,j_1=1}^{n_1}, \end{aligned}$$

or equivalently

$$\begin{aligned} \mathrm {T}_N^{(2)}(f)=\sum \limits _{\left|j_1\right|\le n_1}\sum \limits _{\left|j_2\right|\le n_2}f_{[j_1,j_2]}J_{n_1}^{[j_1]}\otimes J_{n_2}^{[j_2]}, \end{aligned}$$

where $J_{n_i}^{[j_i]}\in \mathbb {R}^{n_i\times n_i}$ are matrices whose entry (*s*, *t*)th equals 1 if $s-t=j_i$ and 0 elsewhere. In other words, a 2-level Toeplitz matrix is a block Toeplitz whose blocks are Toeplitz. When $d=1$, we simplify the notation using

$$\begin{aligned} \mathrm {T}_N(f):=\mathrm {T}_N^{(1)}(f). \end{aligned}$$

### Space–time discretizations: CN-WSGD and BDF2-WSGD

In the following, we briefly review the finite difference space-discretization of problem (1) obtained using the second order accurate Weighted and Shifted Grünwald Difference (WSGD) scheme [23] combined with either Crank–Nicolson (CN) or second-order backward-difference formula (BDF2) schemes in time.

Let $N,M\in \mathbb {N}$ and consider the following uniform space–time grid

$$\begin{aligned} x_i=a+i\varDelta x,\quad \varDelta x=\frac{b-a}{N+1},\quad t^m=m\varDelta t,\quad \varDelta t=\frac{T}{M}. \end{aligned}$$

According to [23], the discretization of $\frac{\partial ^{\alpha }}{\partial _+ x^\alpha }$ yields a lower Hessenberg Toeplitz matrix $A_N^\alpha = \mathrm {T}_N(f_\alpha )$, where

$$\begin{aligned} f_\alpha (x)=\sum \limits _{k=0}^{\infty }\omega _k^{(\alpha )}\mathrm {e}^{\mathrm {i}(k-1)x}, \end{aligned}$$

with

$$\begin{aligned} \omega _0^{(\alpha )}=\frac{\alpha }{2}g_0^{(\alpha )}, \quad \omega _k^{(\alpha )}=\frac{\alpha }{2}g_k^{(\alpha )}+\frac{2-\alpha }{2}g_{k-1}^{(\alpha )},\ \ k\ge 1, \end{aligned}$$

and $g_k^{(\alpha )}=(-1)^k{\alpha \atopwithdelims (){k}}$. A similar reasoning shows that the discretization of $\frac{\partial ^{\alpha }}{\partial _- x^\alpha }$ yields an upper Hessenberg Toeplitz matrix, which coincides with $A_N^T$.

The discretization of the forcing term returns vector $v^m=[v(x_i,t^m)]_{i=1}^N$ and the application of CN and BDF2 schemes in time gives the following linear systems

2 $$\begin{aligned} \left( I_N-rA_{x,N}\right) u^m&=\left( I_N+rA_{x,N}\right) u^{m-1}+\frac{\varDelta t}{2}( v^{m}+v^{m-1} ), \end{aligned}$$

3 $$\begin{aligned} \left( I_N-\frac{4}{3}rA_{x,N}\right) u^m&=\frac{4}{3} u^{m-1}-\frac{1}{3} u^{m-2}+\frac{2}{3}\varDelta tv^m, \end{aligned}$$

respectively, where $r=\frac{\varDelta t}{2\varDelta x^\alpha }$ and

$$\begin{aligned} A_{x,N}=d_+ A_N^\alpha +d_- {A_N^\alpha }^\mathrm {T}. \end{aligned}$$

#### Remark 1

In the case of BDF2, the solution $u^1$ at time $t^1$ is computed with CN and outside the all-at-once linear system. Note that any other one step method could be used to compute $u^1$. For example, although Implicit Euler is only first order accurate, if we only use it once, it will not compromise the global second-order accuracy. Such a statement can be found in [24].

The following proposition, which plays an important role in the definition of the projectors for our multigrid strategy (see Sect. 5), defines the symbol of the spatial discretization.

#### Proposition 1

*Let* $d_\pm =d$, *then* $A_{x,N}=d\cdot \mathrm {T}_N(g_\alpha )$, *where* $g_\alpha (x)=f_\alpha (x)+\overline{f}_\alpha (x)$ *is non-positive and has a zero of order* $\alpha$ at $x=0$.

## Stability of the BDF2-WSGD scheme

In [23] the authors proved the unconditional stability of the CN-WSGD scheme (2), in the constant diffusion coefficients case, as a consequence of the following theorem.

### Theorem 1

[23] *Let* $\lambda$ *be an eigenvalue of* $A_{x,N}$, *then* $\mathrm {Re}(\lambda )<0,\ \forall \alpha \in (1,2)$.

Indeed, in case of a one step scheme like CN, the stability relies on the spectral radius of the iterations matrix of the time stepping algorithm, which is required to be lower than 1. We now aim to prove the stability of the BDF2-WSGD scheme. After the discretization in space we obtain an equation of the form

4 $$\begin{aligned} \frac{\mathrm {d}}{\mathrm {d}t}\mathbf {u}(t)=A_{x,N}\mathbf {u}(t)+\mathbf {v}(t), \end{aligned}$$

where $\mathbf {u}(t),\mathbf {v}(t)$ are, respectively, the semi-discrete in space unknown and forcing term.

The region of absolute stability of a linear multistep method is defined as follows.

### Definition 2

(*Absolute stability region*) The region of absolute stability for the linear multistep method

$$\begin{aligned} \sum _{j=0}^s\alpha _j\mathbf {u}^{n+j}=\lambda \varDelta t\sum _{j=0}^s\beta _j\mathbf {u}^{n+j}, \end{aligned}$$

is the set of points $z\in \mathbb {C}$ for which the roots $\lbrace \zeta _j\rbrace _{j=1}^s$ of the polynomial

5 $$\begin{aligned} \pi (\zeta ,z)=\sum _{j=0}^s\left( \alpha _j-z\beta _j\right) \zeta ^j,\quad z=\lambda \varDelta t, \end{aligned}$$

satisfies the following root conditions:

- $$\left|\zeta _j\right|\le 1$$, for $$j=1,\ldots ,s$$,
- if $\zeta _j$ is a repeated root, then $\left|\zeta _j\right|<1$.

Therefore, again as a consequence of Theorem 1, the following theorem holds.

### Theorem 2

*Let* $\alpha \in (1,2)$ *and consider* $A_{x,N}$ *to be diagonalizable, then BDF2-WSGD scheme* (3) *is unconditionally stable.*

### Proof

Since $A_{x,N}$ is diagonalizable there exists an invertible matrix *V* such that $A_{x,N}=V^{-1}\varLambda V$, where $\varLambda$ is the diagonal matrix containing the eigenvalues of $A_{x,N}$. Therefore, by introducing $\tilde{u}=Vu$, Eq. (3) can be written as *N* uncoupled equations with respect to $\tilde{u}^m$:

$$\begin{aligned} \left( I_N-\frac{4}{3} r\varLambda \right) \tilde{u}^m=\frac{4}{3} \tilde{u}^{m-1} -\frac{1}{3}\tilde{u}^{m-2}+\frac{2}{3}\varDelta t V v^{m}. \end{aligned}$$

Let us fix a row index *i*, then, by definition of *r*,

$$\begin{aligned} \left( 1-{\frac{2}{3}\frac{\varDelta t}{\varDelta x^\alpha }} \lambda _i\right) \tilde{u}_i^m-\frac{4}{3}\tilde{u}_i^{m-1}+\frac{1}{3}\tilde{u}_i^{m-2}=\frac{2}{3}\varDelta t (Vv^m)_i, \end{aligned}$$

which, in the polynomial form of Eq. (5), becomes

$$\begin{aligned} \pi (\zeta ,z)&=\left( 1-\frac{2z}{3\varDelta x^\alpha }\right) \zeta ^2-\frac{4}{3}\zeta +\frac{1}{3}. \end{aligned}$$

By defining $\tilde{z}:=1-\frac{2z}{3\varDelta x^\alpha }$, which is a complex number and can be written as ${\tilde{z}}=a+\text {i}b$, it follows that the roots are

$$\begin{aligned} \left|\zeta _{1,2}\right|=\left|\frac{\frac{4}{3}\pm \sqrt{\frac{16}{9}-\frac{4}{3}{\tilde{z}}}}{2{\tilde{z}}}\right|\le \frac{\left|2 + 2\sqrt{1-\frac{3}{4}{\tilde{z}}}\right|}{3\left|{\tilde{z}}\right|}=\frac{\left|2 + 2\sqrt{1-\frac{3}{4}(a+\mathrm {i} b)}\right|}{3\sqrt{a^2+b^2}}=:g(a,b). \end{aligned}$$

From Theorem 1, we have that $\text {Re}(\tilde{z})=a>1$ for $\alpha \in (1,2)$, and the study of the maximum of function *g*(*a*, *b*) shows that $\mathop {\sup }\nolimits _{a>1}\ g(a,b)<1$. $\square$

The following corollary exploits the density of diagonalizable matrices into the space of square matrices to remove the diagonalizability hypothesis in Theorem 2.

### Corollary 1

*Let* $\alpha \in (1,2)$, *then BDF2-WSGD scheme* (3) *is unconditionally stable.*

### Proof

Let us suppose that $A_{x,N}$ is not diagonalizable, otherwise the thesis follows from Theorem 2. Let us consider the Schur decomposition $QTQ^\mathrm {H}$ of $A_{x,N}$, where *Q* and *T* are unitary and upper triangular matrices, respectively. Note that due to the structure of *T*, its diagonal elements are the eigenvalues of *T* and that, by similarity, they coincide with the eigenvalues of $A_{x,N}$.

Since we are assuming that $A_{x,N}$ is not diagonalizable, *T* has at least two diagonal elements that are equal. Let us then consider matrix $B_N=Q\tilde{T}Q^\mathrm {H}$, where $\tilde{T}$ is obtained from *T* by properly shifting its diagonal entries such that $\tilde{T}$ becomes diagonalizable. More precisely, for $\epsilon >0$ and for $i=1,\ldots ,N$ the *i*th diagonal element of $\tilde{T}$ is $\tilde{t}_{ii}=t_{ii}-\delta _i$ with $0\le \delta _i<\epsilon$ such that $\tilde{t}_{ii}\ne \tilde{t}_{jj},\ \forall i,j=1,\ldots ,N$ and $i\ne j$.

Since $B_N$ is diagonalizable and its eigenvalues have negative real part, Theorem 2 applies to $B_N$, and since

$$\begin{aligned} \left\Vert B_N-A_{x,N}\right\Vert _2=\left\Vert \tilde{T}-T\right\Vert _2=\max _{i=1,\ldots ,N}\delta _i<\epsilon , \end{aligned}$$

by letting $\epsilon \rightarrow 0$ the thesis is proven. $\square$

### Remark 2

The unconditional stability of BDF2 combined with a central finite difference scheme for discretizing the fractional derivative operator, was given in [15]. Therein, the diffusion coefficients were both equal to 1. Under the diagonalizability hypothesis, Theorem 2 extends the unconditional stability of BDF2 to the case where the space scheme is WSGD and the diffusion coefficients are not necessarily equal to each other. Corollary 1 generalizes Theorem 2 to the case of a non diagonalizable matrix $A_{x,N}$.

## All-at-once rephrasing of our problem and related spectral study

Starting from Eqs. (2) and (3), and chaining the unknown $u^{m}$ at each time step into a unique vector as

$$\begin{aligned} u_{\text {CN}}=[u^{1},\ldots ,u^{M}]^\mathrm {T}\in \mathbb {R}^{NM},\ u_{\text {BDF2}}=[u^{2},\ldots ,u^{M}]^\mathrm {T}\in \mathbb {R}^{N(M-1)}, \end{aligned}$$

in the case of CN and BDF2, respectively, we can rephrase the original discretized problem as the following large block linear systems

6 $$\begin{aligned} A_{\text {S}}u_\text {S}=b_{\text {S}},\ \text {S}\in \lbrace \text {CN,BDF2}\rbrace , \end{aligned}$$

where

$$\begin{aligned} A_{\text {CN}}&=\begin{pmatrix} I-rA_{x,N} &{} 0 &{} 0 &{} 0 \\ -I-rA_{x,N} &{} I-rA_{x,N}&{} 0 &{} 0 \\ 0 &{} \ddots &{} \ddots &{} 0 \\ 0 &{} 0 &{} -I-rA_{x,N}&{}I-rA_{x,N}\end{pmatrix},\\ A_{\text {BDF2}}&=\begin{pmatrix} I-\frac{4}{3} rA_{x,N} &{} 0 &{} 0 &{} 0 &{} 0\\ -\frac{4}{3}I &{} I-\frac{4}{3}rA_{x,N}&{} 0 &{} 0 &{} 0\\ \frac{1}{3}I &{}-\frac{4}{3}I &{} I-\frac{4}{3}rA_{x,N}&{} 0 &{} 0 \\ 0 &{} \ddots &{} \ddots &{} \ddots &{} 0\\ 0 &{} 0 &{} \frac{1}{3}I &{} -\frac{4}{3}I &{}I-\frac{4}{3}rA_{x,N}\\ \end{pmatrix}, \end{aligned}$$

and

$$\begin{aligned} \ b_{\text {CN}}=\begin{pmatrix} (I+rA_{x,N})u^0+\varDelta tv^{1-\frac{1}{2}}\\ \varDelta tv^{2-\frac{1}{2}}\\ \vdots \\ \varDelta tv^{M-\frac{1}{2}} \end{pmatrix}, \qquad b_{\text {BDF2}}=\begin{pmatrix} \frac{4}{3}u^1-\frac{1}{3}u^0+\frac{2}{3}\varDelta tv^{2}\\ -\frac{1}{3}u^1+\frac{2}{3}\varDelta tv^{3}\\ \frac{2}{3}\varDelta tv^{4}\\ \vdots \\ \frac{2}{3}\varDelta tv^{M} \end{pmatrix}. \end{aligned}$$

Note that both coefficient matrices $A_{\text {CN}}$, $A_{\text {BDF2}}$ are two-level Toeplitz according to Definition 1, and hence we can compute their symbol.

From Proposition 1 we recall that $A_{x,N}=d\cdot \text{T}_N(g_\alpha )$, where $g_\alpha (x)$ has a zero of order $\alpha$ at $x=0$. Then, by assuming $d_\pm =d$ and $N,M\rightarrow \infty$, we have

$$\begin{aligned} A_{\text {S}}=\text {T}^{(2)}_{N M}(h_{\text {S}}),\ \text {S}\in \lbrace \text {CN,BDF2}\rbrace , \end{aligned}$$

with $h_\text {S}$ defined as follows:

(i) if $r$ is constant, then
  7 $$\begin{aligned} \begin{aligned} \bullet&\quad h_{\text {CN}}(x,t)=1-\text {e}^{\text {i}t}-d\cdot rg_\alpha (x)\left( 1+\text {e}^{\text {i}t}\right) ,\\ \bullet&\quad h_{\text {BDF2}}(x,t)=1-\frac{4}{3}\mathrm {e}^{\text {i}t}+\frac{1}{3}\mathrm {e}^{2\text {i}t}- \frac{4}{3}d \cdot rg_\alpha (x), \end{aligned} \end{aligned}$$
  and both functions have a unique zero at $(x,t)=(0,0)$ of order 1 and α in *t* and *x*, respectively;
(ii) if $r\rightarrow 0$, then from (7) we have
  $$\begin{aligned} \bullet&\quad h_{\text {CN}}(x,t)=1-\mathrm {e}^{\text {i}t},\\ \bullet&\quad h_{\text {BDF2}}(x,t)=1-\frac{4}{3}\mathrm {e}^{\text {i}t}+\frac{1}{3}\mathrm {e}^{2\text {i}t}, \end{aligned}$$
  and both functions have a zero of order 1 at $t=0,\ \forall x$;
(iii) if $r\rightarrow \infty$, by grouping up *r* in (7), we have
  $$\begin{aligned} \bullet&\quad h_{\text {CN}}(x,t)=-d(1+\mathrm {e}^{\text {i}t})g_\alpha (x),\\ \bullet&\quad h_{\text {BDF2}}(x,t)= -\frac{4}{3}d\cdot g_\alpha (x), \end{aligned}$$
  where $h_{\text {CN}}$ has a zero of order 1 at $t=\pi ,\ \forall x$ and a zero of order $\alpha$ at $x=0,\ \forall t$, while $h_{\text {BDF2}}$ vanishes only at $x=0,\ \forall t$ with order $\alpha$.

The presence of at least a line of zeros in the symbol is called *anisotropy*. The latter becomes stronger as the number of such lines increases. We expect then case (iii) for CN to be much harder to be numerically treated than all other cases.

### Remark 3

If we suppose *r* to be constant and let $d\rightarrow \infty$ or $d\rightarrow 0$ then the same results as in case (ii) and (iii), with *r* in place of *d*, hold. In practice, since *r*, *d* are fixed coefficients, the anisotropy arises when $d\cdot r$ is very large or very small.

### Remark 4

The study of the symbol $h_\text {S}$ can easily be extended to the case where $d_+\ne d_-$ using the results in [6].

## Multigrid methods for all-at-once systems

This section is devoted to the design of multigrid strategies based on the spectral study performed in Sect. 4 for the linear systems in (6). With this aim, we first recall the basics of the multigrid in Sect. 5.1, then we discuss our multigrid proposals in Sect. 5.2.

### Multigrid idea and convergence results

Multigrid methods, introduced in [22], combine two iterative methods known as smoother and coarse grid correction (CGC). Given the linear system $A_Nx=b$, $A_N\in {\mathbb {R}}^{N\times N}$, the former is typically a stationary iterative method, which we denote with $\mathcal {S}^{\nu }\left( x,A_N,b\right)$, where $\nu$ is the number of iterations.

To give a precise account of what the CGC is, let us consider the most basic version of multigrid, often used for proving convergence results, i.e., the two-grid method (TGM). Given a full-rank matrix $P_N\in {\mathbb {R}}^{N\times k}$, with $k<N$, a step of TGM is defined by Algorithm 1. 
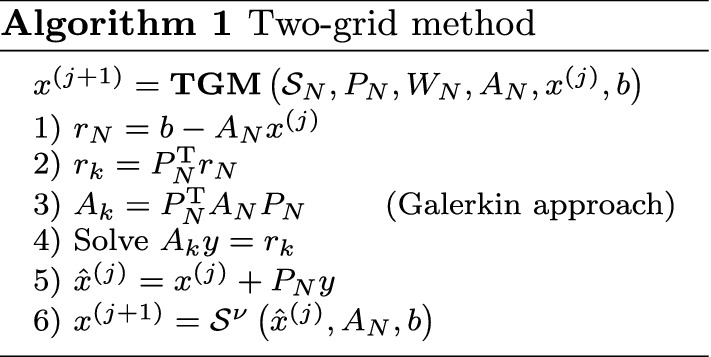


Steps 1) to 5) define the CGC and step 6) is called post-smoother. To strengthen the algorithm, a second smoother, which is called pre-smoother, could be added before the CGC. V-Cycle is obtained from TGM by replacing Step 4) with a recursive call of TGM. The Garlerkin approach is useful for the convergence analysis, but in practice it could be too computationally expensive. Therefore, it is often replaced by a geometric approach, which consists in the rediscretization of the equation over a coarser grid. This leads to a less robust algorithm, but allows to maintain the same structure of the coefficient matrix at each level and is highly parallelizable.

Moreover, if the matrix $A_N$ shows a block Toeplitz-like structure, by choosing the geometric approach the matrix–vector products can be performed by means of the fast Fourier transform algorithm at each coarser grid.

Let $K_N\in \mathbb {R}^{N\times {\lfloor }{\frac{N}{2}}{\rfloor }}$ be the down-sampling matrix, which keeps an entry every two, then the one-dimensional grid transfer operator $P_N$ is defined as

$$\begin{aligned} P_N=K_N\mathrm {T}_N(p). \end{aligned}$$

In more than one dimension, the projector is defined through the tensor product. For instance, in two dimensions the required projector $P_{NM}$ is defined as

$$\begin{aligned} P_{NM}&=\left( K_M\otimes K_N\right) \mathrm {T}^{(2)}_{NM}(p_1(x)p_2(t))\\&=\left( K_M\otimes K_N\right) \left( \mathrm {T}_M(p_2(t))\otimes \mathrm {T}_N(p_1(x))\right) \\&=P_{t,M}\otimes P_{x,N}, \end{aligned}$$

where

$$\begin{aligned} P_{x,N}=K_N\mathrm {T}_N(p_1), \quad P_{t,M}=K_M\mathrm {T}_M(p_2), \end{aligned}$$

are the projectors in space and time generated by $p_1(x)$ and $p_2(t)$, respectively. Notice, that this is the case we are interested in. Indeed, by considering the time as an additional dimension, we have reinterpreted the original one-dimensional problem as a two-dimensional one.

The convergence of the V-cycle, proved in [1], requires the following condition on $p(x,t)=p_1(x)p_2(t)$. Let $f\ge 0$ be the symbol of $A_N$, vanishing only at $(x_0,t_0)$, then *p* has to satisfy

8 $$\begin{aligned} \limsup \limits _{{(x,t)}\rightarrow (x_0,t_0)}\frac{p({\hat{x}},{\hat{t}})}{f(x,t)}=c<+\infty , ~~~~~~~~~~\forall ({\hat{x}},{\hat{t}})\in \mathscr {M}(x,t), \end{aligned}$$

where $\mathscr {M}(x,t)=\{(x,\pi -t),(\pi -x,t),(\pi -x,\pi -t)\}$ is the set of the “mirror points" of (*x*, *t*).

In the case where the symbol *f* has a whole line of zeros along the axes, relation (8) does not hold anymore. In such a case, an efficient alternative to standard *V*-cycle is given by the so-called *semi-coarsening* [8]. The projector in the semi-coarsening approach is defined by considering the vanishing variable as a parameter, and then building the projector in the remaining variable according to the one-dimensional version of relation (8). The projector in the other dimension is simply given by an identity matrix.

### Multigrid methods for the all-at-once systems

We now see how the results recalled in Sect. 5.1 apply to our case. In particular, we define the projector according to condition (8) and the properties of $h_\text {S}$ defined in Sect. 4. Let us first introduce the polynomials

$$\begin{aligned} p_1(x)=2+2\cos (x),\quad p_{2}^+(t)=1+\mathrm {e}^{ \text {i}t},\quad p_{2}^-(t)=1-\mathrm {e}^{\text {i}t}. \end{aligned}$$

Note that:

- $p_1(x)$ has a zero of order 2 at $x=\pi$;
- $p_{2}^+(t)$ has a zero of order 1 at $t=\pi$;
- $p_{2}^-(t)$ has a zero of order 1 at $t=0$.

In the case of CN, according to the analysis in Sect. 4, we distinguish the following three cases:

1. If *r* is constant, then $h_{\text {CN}}$ has a unique zero of minimum order 1 at $(x,t)=(0,0)$. The mirror points of $(x,t)=(0,0)$ are $(0,\pi ),(\pi ,0),(\pi ,\pi )$. Since $p_1(x)$ and $p_{2}^+(t)$ vanish at $x=\pi$ and $t=\pi$, respectively, $p(x,t)=p_1(x)p_{2}^+(t)$ vanishes at the mirror points with a minimum order of 1 and hence satisfies relation (8).
2. In the anisotropic case where $r\rightarrow 0$, $h_{\text {CN}}$ is zero on the whole *x*-axis, then we opt for semi-coarsening in time. Precisely, by considering variable *x* as a parameter, $h_{\text {CN}}$ has a unique zero of order 1 at $t=0$. Then, we generate $P_{t,M}$ through $p_{2}^+(t)$ that has a zero of order 1 at the mirror point $t=\pi$. The projector $P_{x,N}$ is given by the identity matrix $I_N$.
3. In the anisotropic case, where $r\rightarrow \infty$, $h_{\text {CN}}$ is zero on both axes. The theory does not apply to this scenario. Nevertheless, as a first attempt to dominate (at least partially) this other kind of anisotropy, we use again standard semi-coarsening in both time and space. Precisely,
  When *x* is considered as a parameter, $h_{\text {CN}}$ has a zero of order 1 at $t=\pi$. Therefore, we perform semi-coarsening in time replacing $P_{x,N}$ with $I_N$ and generating $P_{t,M}$ through $p_{2}^-(t)$, whose first order zero at $t=0$ satisfies relation (8) for *x* fixed, i.e., considering only the one-dimensional problem in the variable *t*.
  When *t* is considered as a parameter, $h_{\text {CN}}$ has a zero of order $\alpha$ at $x=0$. Therefore, the semi-coarsening in space is defined by replacing $P_{t,M}$ with the identity matrix $I_M$ and generating $P_{x,N}$ through $p_1(x)$, which has a zero of order 2 at the mirror point $x=\pi$, again according to condition (8) for *t* fixed.

In the case of BDF2, items 1) and 2) are identical. Regarding item 3), i.e. when $r\rightarrow \infty$, symbol $h_{\text {BDF2}}$ vanishes only over the line $x=0,\ \forall t$ with order $\alpha$. Hence we have a standard anisotropy, like in item 2), but this time along the *t*-axis. Therefore, we consider a semi-coarsening in space by setting $P_{t,M}=I_M$ and by generating $P_{x,N}$ through $p_1(x)$, whose position and order of the zero satisfies relation (8) for *t* fixed.

Concerning the *smoother*, we consider $\omega$-weighted block Jacobi method ($\omega$-BJ), where the diagonal blocks are of the form $I-\xi r A_{x,N}$ with $\xi=1$ for CN and $\xi=4/3$ for BDF2. The reason for such a choice is that it is parallelizable and it allows to exploit the structure of the coefficient matrix. Moreover $\omega$-BJ converges for any $\omega \in (0,1]$ whenever the blocks are of size $N\times N$, hence the study of its relaxation parameter is only related to the smoothing property along the time axis (see Sect. 6.1 for a numerical discussion about this issue). In our tests, the inversion of the blocks in block-Jacobi is performed through the Matlab function backslash.

We stress that, choosing standard Jacobi would decrease the computational cost and would also exploit the structure of the coefficient matrix, but its use would ask for a tougher study of the relaxation parameter in order to ensure the convergence.

#### Remark 5

Note that in case of BDF2, when $d\rightarrow \infty \text { or }r\rightarrow \infty$, the coefficient matrix in Eq. (6) tends to a block diagonal Toeplitz matrix. This means that, when $d\cdot r$ becomes large, using the multigrid is pointless since its smoother is already computing the solution accurately enough.

## Numerical results

In this section we investigate the performances of $\omega$-BJ, two-grid method (TGM) and V-Cycle (V), mainly used as standalone solvers for solving the linear system in (6). Few numerical results concerning the use of both TGM and V as preconditioners (only one iteration) for GMRES are also given.

Both TGM and V-Cycle will have one iteration of $\omega$-BJ as post-smoother and no pre-smoothing iteration (the reason is given in Sect. 6.1). In V-Cycle we halt the coarsening at the 5th level, when the coefficient matrix has a minimum size of $N_5\times M_5$ with $N_5\ge \frac{N}{2^5}$ and $M_5\ge \frac{N}{2^5}$, depending on the coarsening technique, and the solution on the coarsest level is performed through the backslash Matlab function, which is a direct solver.

As already clarified in Remark 1, the solution $u^1$ at the first time step is computed outside the coefficient matrix. One could of course include the computation of $u^1$ in the coefficient matrix as done in [11]. In our case, due to the computationally expensive smoother we use, the difference between the two approaches is negligible. A comparison between the two approaches in terms of iterations can be found in Sect. 6.6.

The section is organized as follows. In the first part we aim at explaining how we fix the fractional derivative order $\alpha$, and the relaxation parameter $\omega$ in our numerical examples. Precisely, in Sect. 6.1 we test the performances of ω-BJ for two different values of $\omega$ and we show that it generates jumps along the time axis, independently of $\omega$. In Sect. 6.2, we check how much TGM is sensitive to $\alpha$, and we numerically prove that its behavior is only slightly $\alpha$-dependent.

Aside from $\alpha$ and $\omega$, we also need to clarify how we choose between the two projector generators $p^+_2$ and $p^-_2$ discussed in Sect. 5.2 when performing semi-coarsening in time for CN. This is the subject of Sect. 6.3. In Sects. 6.4–6.5 we perform few tests with large *N*, *M* to numerically check the robustness of TGM and V as *N*, *M* increase in both constant and variable diffusion coefficients cases. Finally, in Sect. 6.6 we provide a two-dimensional example.

All our tests have been run on a server with Intel(R) Xeon(R) Silver 4114 at 2.20GHz with Matlab 2019b. For all methods we fix the tolerance to $10^{-7}$ and the initial guess as the null vector. We use the built-in gmres function, whose preconditioner is left-sided. For this reason we force GMRES to reach the required tolerance on the actual residual through a ‘by hand’ restart. A right preconditioned GMRES could be of course employed and it would basically give the same amount of iterations.

*Notation.* In the following, we denote with TGMp (resp. Vp), p$\in \lbrace \text {x},\text {t},\text {xt}\rbrace$ the TGM (resp. V) that uses ω-BJ as post-smoother and performs semi-coarsening in space ($\mathrm{p}=\mathrm{x}$), time ($\mathrm{p}=\mathrm{t}$), or both space and time ($\mathrm{p}=\mathrm{xt}$). Precisely:

- ‘x’ denotes the space semi-coarsening, whose projector is generated by $p_1(x)$;
- ‘t$_\pm$’ denotes the time semi-coarsening, whose projector is generated by $p_2(t)^\pm$;
- ‘xt$_\pm$’ denotes the full-coarsening, whose projector is generated by $p_1(x)p_2(t)^\pm$.

The addition of ‘(G)’ after the solver name stands for ‘Galerkin approach’. In case nothing is specified, geometric approach is adopted. Finally, the presence of ‘($\mathcal {P}$)’ in the name of the solver means that the considered multigrid method is set as GMRES preconditioner.

We point out that due to space limitations, in the key of each figure we omit the name of the method and specify only the projector. For instance, we write simply xt$_+$ in place of TGMxt$_+$. The name of the method will be clear from the caption of the figure.

All the results contained in the Sects. 6.1–6.4 refer to the following example.

### Example 1

In this example we assume the diffusion coefficients to be constant and equal, that is $d_\pm =d$. The space and time domains in problem (1) are fixed as $\varOmega =(0,2)$, and [0, 1] respectively, while the true solution and the solution at $t=0$ are given by

$$\begin{aligned} u_{ex}(x,t)=4\mathrm {e}^{-t} x^2(2-x)^2, \qquad u_0(x)=4x^2(2-x)^2. \end{aligned}$$

The numerical approximation of *v* is computed starting from the discretized exact solution.

### Behavior of ω-BJ smoother

Here we test the “smoothing properties” of $\omega$-BJ. Let us consider $\alpha =1.5$, $d=1$, $N=63$ and, to better point out the behavior of ω-BJ along the time axis, we fix $M=7\ll N$.

**Fig. 1:**
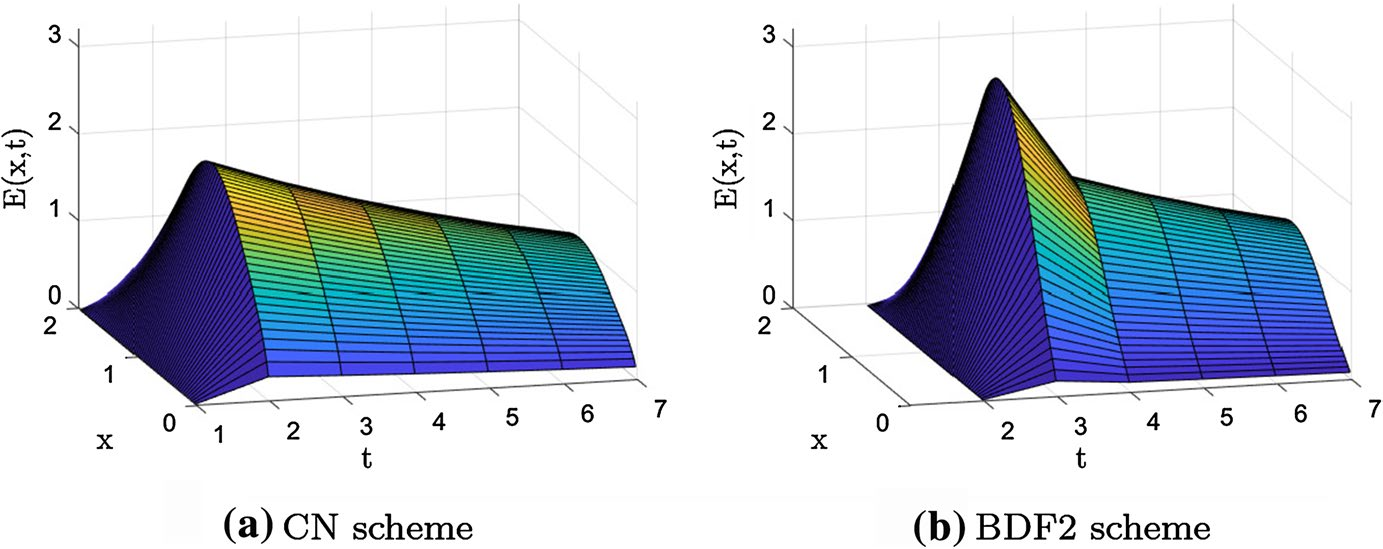
Example 1—Error *E*(*x*, *t*) after one iteration of 1-BJ

Figure 1 shows the error, reshaped as a space–time surface, after one iteration of $\omega$-BJ for both linear systems in Eq. (6).

**Fig. 2:**
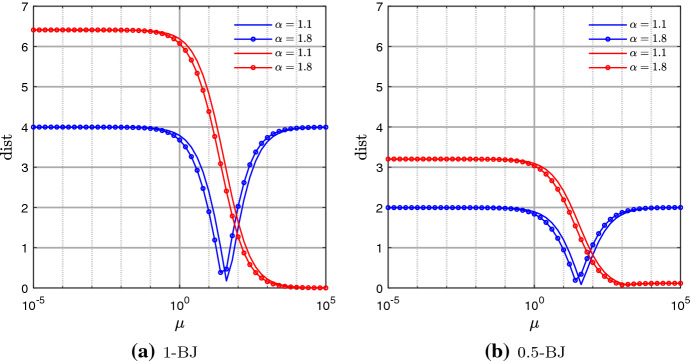
Example 1—Vertical displacement $\text {dist}(E)$, in Eq. (9), after 1 iteration of ω-BJ when using both CN (blue) and BDF2 (red)

We note that, in case of CN, one iteration of 1-BJ generates a jump along the time axis from $t^1$ to $t^2$. In Fig. 1b, where the 2-step method BDF2 is considered, such a jump involves also $t^3$ due to the longer stencil of BDF2 with respect to CN. On the other hand, both surfaces in Fig. 1 do not show any jump along the *x*-axis.

We now analyze the jump in time varying the magnitude of $\mu :=d\cdot r$, where *r* is the grid dependent scale parameter. We introduce the function

9 $$\begin{aligned} \text {dist}(E)=|E(1,t^2)-E(1,t^1)|+|E(1,t^3)-E(1,t^2)|, \end{aligned}$$

which measures the vertical displacement of the discrete error *E*(*x*, *t*) in the first three time steps at the midpoint $x=1$. We note that $\text {dist}(E)=0$ if and only if *E* is constant in the first three time steps. In other words, as far as $\text {dist}(E)\approx 0$, *E* is smooth, and this indicates that $\omega$-BJ is a good smoother.

Figures 2a and b show how $\text {dist}(E)$ behaves for both CN and BDF2, fixed $N=M=63$, $\omega =1,0.5$, $\alpha =1.1, 1.8$, and varying $\mu \in [10^{-5},10^5]$. As we can see, both discretizations are characterized by a region where the jump is negligible. In detail, the jump generated in CN is negligible only when $\mu \approx 10$. In BDF2, instead, the jump becomes negligible as $\mu$ increases. Moreover, for both CN and BDF2, the jump slightly moves while varying $\alpha$, and it halves its magnitude when switching from $\omega =1$ to $\omega =0.5$.

In summary, in all the considered cases $\omega$-BJ generates jumps along the time axis which means that the projection along such axis could be inaccurate. In order to face this drawback, in the following we only consider $\omega$-BJ as post-smoother avoiding pre-smoothing iteration at the first iteration. This choice is supported by the idea that applying the CGC before the smoother could reduce the jump, preventing then the projection of a non-smooth error.

### Behavior of TGM varying $\alpha$

In Sect. 6.1, we observed that $\text {dist}(E)$, in Eq. (9), slightly varies with $\alpha$. This could lead to a difference in the behavior of the multigrid depending on $\alpha$, when solving the two linear systems in (6). Here we perform few tests which show that the behavior of the proposed TGM is almost independent of $\alpha$ and hence that justify the choice of a fixed value for $\alpha$ in the reminder of the numerical tests.

**Fig. 3:**
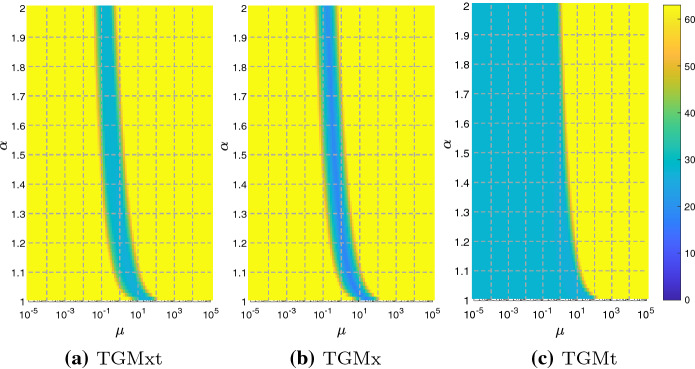
Example 1 - Iterations to tolerance varying $\alpha$ and $\mu$, fixed $\omega =0.5$, and using CN scheme

**Fig. 4:**
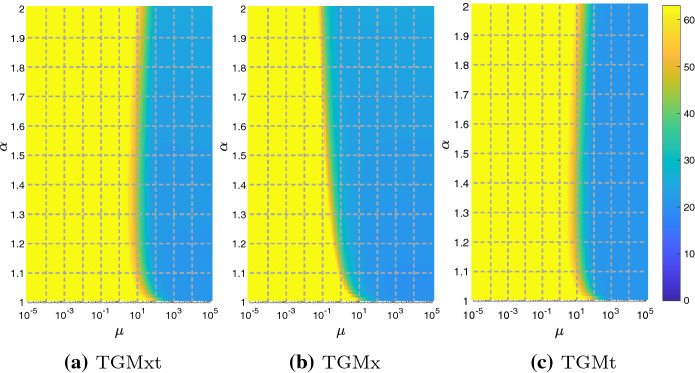
Example 1—Iterations to tolerance varying $\alpha$ and $\mu$, fixed $\omega =0.5$, and using BDF2 scheme

In Figs. 3 and 4, we check the number of iterations of TGMx, TGMt, and TGMxt, with fixed $N=M=2^6-1$, and varying $\mu \in [10^{-5},10^{5}]$, and $\alpha \in (1,2]$. Concerning the choice of $\omega$ in the $\omega$-BJ smoother, several tests (not reported here because of space limitations) show that, in the case of CN, the choice of $\omega =1$ causes bad convergence results for both TGMxt and TGMt. On the other hand, $\omega =0.5$ provides a good convergence, according also to the analysis in Sect. 6.1, for both CN and BDF2. Therefore, in the rest of this section we fix $\omega =0.5$. We stress that such discussion on the relaxation parameter is not intended as a substitute of a rigorous study, and that a theoretical approach to the subject will be investigated in a future work.

Figure 3, where we use CN scheme, shows that by increasing $\alpha$ the optimal region of convergence (blue) shifts to the left for any of the considered algorithms. Regarding BDF2, instead, Fig. 4 shows that the blue region shifts to the left as $\alpha$ increases only in the case of TGMx. In the other two cases, their number of iterations stays almost independent of $\alpha$.

Summarizing, the width of the blue regions does not seem to significantly change while varying $\alpha$. Therefore, in the following we restrict our analysis to the case where $\alpha =1.5$.

### Time projection performances for the CN scheme

In Sect. 4 we have shown that the symbol $h_\text {CN}$ has a zero in *t* that moves from 0 to $\pi$ depending on how *r* or *d* behave asymptotically, and then on the magnitude of $\mu =d\cdot r$. As discussed in Sect. 5.2, this means that the projector in time must change as well from t$_+$ to t$_-$ according to $\mu$. Here, we show that the latter does not work satisfactorily in practical applications when $\mu$ is large.

**Fig. 5:**
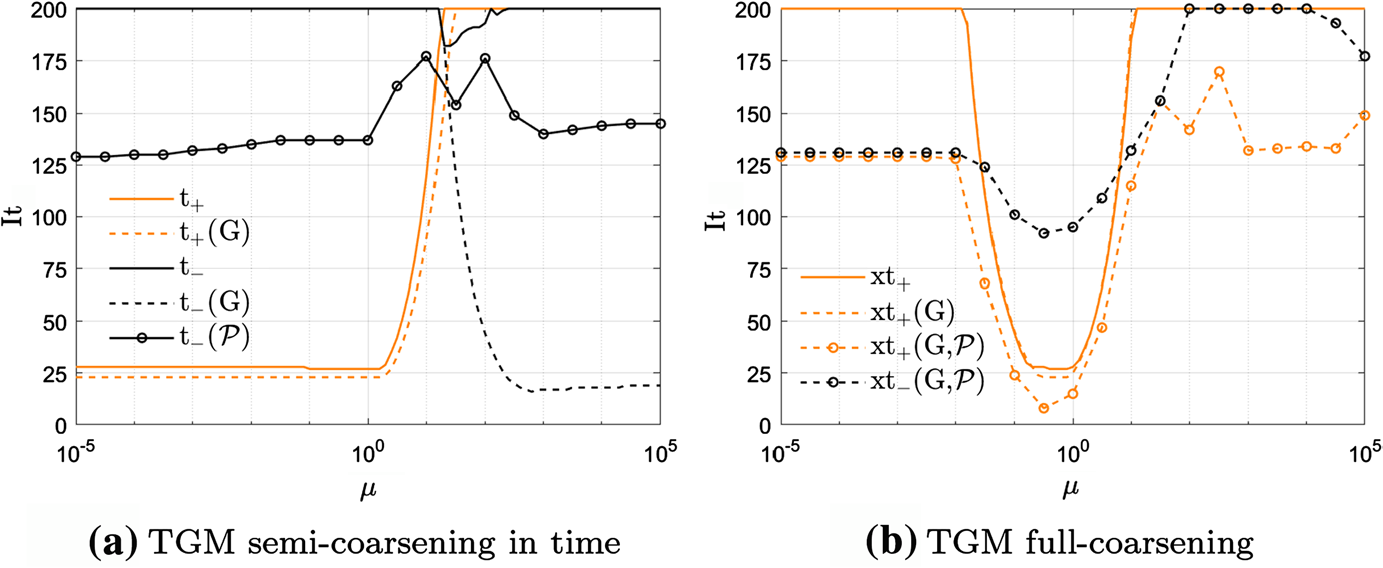
Example 1—time projectors performances for the CN scheme

Let us fix $\alpha =1.5$ and $N=M=2^7-1$. Figure 5a shows the iterations to tolerance of TGMt$_{\pm }$, TGMt$_{\pm }$(G), TGMt$_{-}$($\mathcal {P}$) while varying the magnitude of $\mu \in [10^{-5},10^5]$. We note, in line with the discussion in Sect. 5.2, that the Galerkin approach allows TGMt$_+$(G) to converge in a low amount of iterations when $\mu \in [10^{-5},1]$, that is for small values of $\mu$. When considering the less robust geometric approach, TGMt$_+$ still yields good convergence results in the same range of $\mu$, even if the iteration number slightly increases.

In the case where $\mu \gg 1$, the only working method is TMGt$_-$(G). Unfortunately, the Galerkin approach is not of practical use since it is too computationally expensive. Regarding the geometric approach, TGMt$_-$ results unpractical also when used as GMRES preconditioner (refer to TGMt$_-$($\mathcal {P}$) in Fig. 5a).

The reason why geometric and Galerkin methods behave differently is due to the large difference between the matrices at the coarser level obtained with the two approaches. Indeed, the convergence condition given in (8) requires the Galerkin approach, which leads to a coarser matrix having a symbol that vanishes at the origin (see [1] for details). Differently, the geometric approach, which consists in discretizing the same problem over a coarser grid, builds a coarser matrix that vanishes again at $t=\pi$ and that shows then opposite spectral behavior with respect to Galerkin.

In Fig. 5b, TGM with full-coarsening is shown not to work in the anisotropic cases $\mu <10^{-1}$ and $\mu >10$, independently of the time projectors and the approach for computing the matrix at the coarser level.

In conclusion, in the following we only consider the time projector given by t$_+$ and we denote it simply with ‘t’, since it is the only projector that allows TGM with both semi-coarsening in time and full-coarsening to yield good convergence results for the geometric approach.

### Comparison between CN and BDF2: TGM and V-cycle performances

Now we discuss how the performances of TGM and V-cycle with both semi- and full-coarsening vary depending on the adopted discretization scheme, i.e., CN or BDF2.

**Fig. 6:**
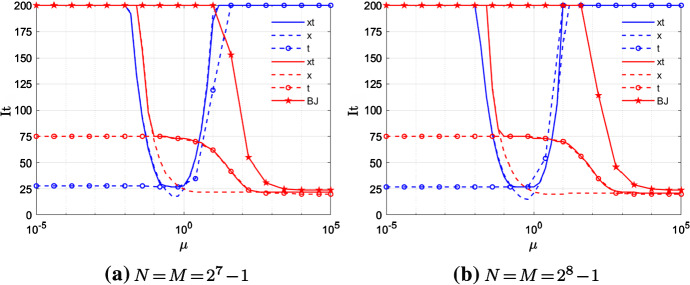
Example 1—TGM performances either using CN scheme (blue) or BDF2 scheme (red), and fixed $\alpha =1.5$

Let us discuss first the behavior of TGM. In Fig. 6, we compare the iterations to tolerance of TGMxt, TGMx, TGMt for both CN and BDF2 varying the magnitude of $\mu \in [10^{-5},10^5]$ and fixed $N=M\in \{2^7-1,2^8-1\}$. In case of BDF2, the iteration number of 1-BJ used as a standalone solver are displayed as well.

We note that, in the case of CN, the iterations to tolerance of TGMt look stable for $\mu <1$ as *N*, *M* increase. The same holds for TGMxt and TGMx when $\mu \approx 1$. Nothing seems to work when $\mu > 10$ again independently of *N*, *M*, which is what we are expecting due to the strong anisotropy of this specific case discussed in Sect. 5.2.

In the case of BDF2, again according to our theoretical analysis, TGMx and TGMt yield good convergence results when $\mu >1$ and $\mu <1$, respectively, and both are stable as *N*, *M* increase. In line with what we observed in Sect. 6.1, the high number of iterations of TGMt, even if constant, could be due to the bad smoothing effects along the time axis of 0.5-BJ. Concerning TGMxt, it yields the same iterations to tolerance as TGMt when $\mu >10^{-1}$. Note that for this example both TGMxt and TGMt work where they are not supposed to, i.e., in the anisotropic case $\mu \gg 1$. This is due to the smoother that, according to Remark 5, is already a robust enough solver.

**Fig. 7:**
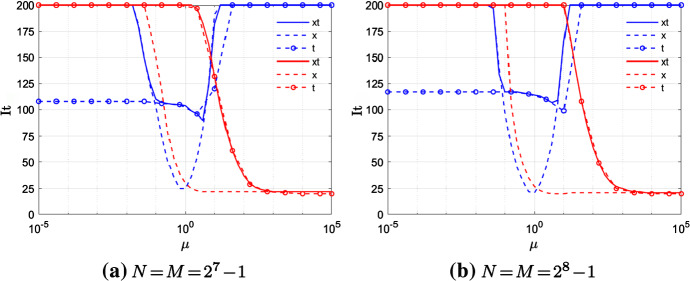
Example 1—V-cycle performances either using CN scheme (blue) or BDF2 scheme (red), and fixed $\alpha =1.5$

Due to the high computational cost of TGM, in Fig. 7 we switch from TGM to V-cycle and we check its behavior depending on the chosen time discretization scheme.

In the case of CN, conversely to the results obtained for TGM, the only projector which seems to allow V-cycle to converge in a reasonable amount of iterations, is the semi-coarsening in space. However, it works in a really small region, i.e. when $\mu \approx 1$, which is close to the region where one iteration of 0.5-BJ yields a smooth solution (go back to Fig. 2).

Regarding BDF2, Vx converges in almost the same amount of iterations as TGMx when $\mu > 1$. In particular, for $\mu \in \left[ 1,10^3\right]$ the block diagonal part of $A_\text {BDF2}$ is not dominant and hence 0.5-BJ has a slow convergence, but when it is used as smoother in Vx we obtain a robust and fast convergent method. Moreover, we note that the region where 0.5-BJ used as standalone solver is already enough robust becomes smaller as the mesh-size increases. This is not the case for Vx, which is then faster than 0.5-BJ in a wider range of $\mu$ as *N*, *M* become large. Concerning Vt and Vxt, their plots are basically superposed independently of $\mu$, and they perform well only for large values of $\mu$ again because of the ω-BJ smoother.

In the case of a semicoarsening in space only, since time interpolation is not involved, larger values of $\omega$ could be used. Tests which are not reported here show a reduction in the iteration number of the multigrid with ω-BJ, with $\omega \approx 1$, when applied to both CN and BDF2 for almost the same values of $\mu$ where it performs well with $\omega =0.5$.

We note that, for both CN and BDF2, the iterations to tolerance of all the tested V-cycles stay almost stable as *N*, *M* increase. Moreover our results are in line with the results reported in Figure 2, Section 4.4 of [12], where multigrid with coloured pointwise Gauss–Seidel as smoother is used to solve a space–time linear system obtained from the discretization of a standard time-dependent diffusion equation.

### A variable diffusion coefficients example

We now consider the example taken from [14] in which the diffusion coefficients are not constant.

#### Example 2

We assume the space and time domains in problem (1) as $\varOmega =(0,2)$, and [0, 1] respectively, and define the diffusion coefficients, the true solution and the solution at $t=0$ as follows

$$\begin{aligned} d_-(x,t)&=d\cdot \varGamma (3-\alpha )x^\alpha ,&d_+(x,t)&=d\cdot \varGamma (3-\alpha )(2-x)^\alpha ,\\ u_{ex}(x,t)&=4\mathrm {e}^{-t} x^2(2-x)^2,&u_0(x)&=4x^2(2-x)^2, \end{aligned}$$

where $\varGamma$ is the gamma function and $d>0$. When $d=1$, the forcing term is given by

$$\begin{aligned} v(x,t)=-32\mathrm {e}^{-t}\left( x^2+\frac{1}{8}((2-x)^2\right) (8+x^2)-\frac{3(x^3+(2-x)^3)}{3-\alpha }+\frac{3(x^4+(2-x)^4))}{(4-\alpha )(3-\alpha )}, \end{aligned}$$

while in the remaining cases, the numerical approximation of *v* is computed starting from the discretized exact solution.

**Fig. 8:**
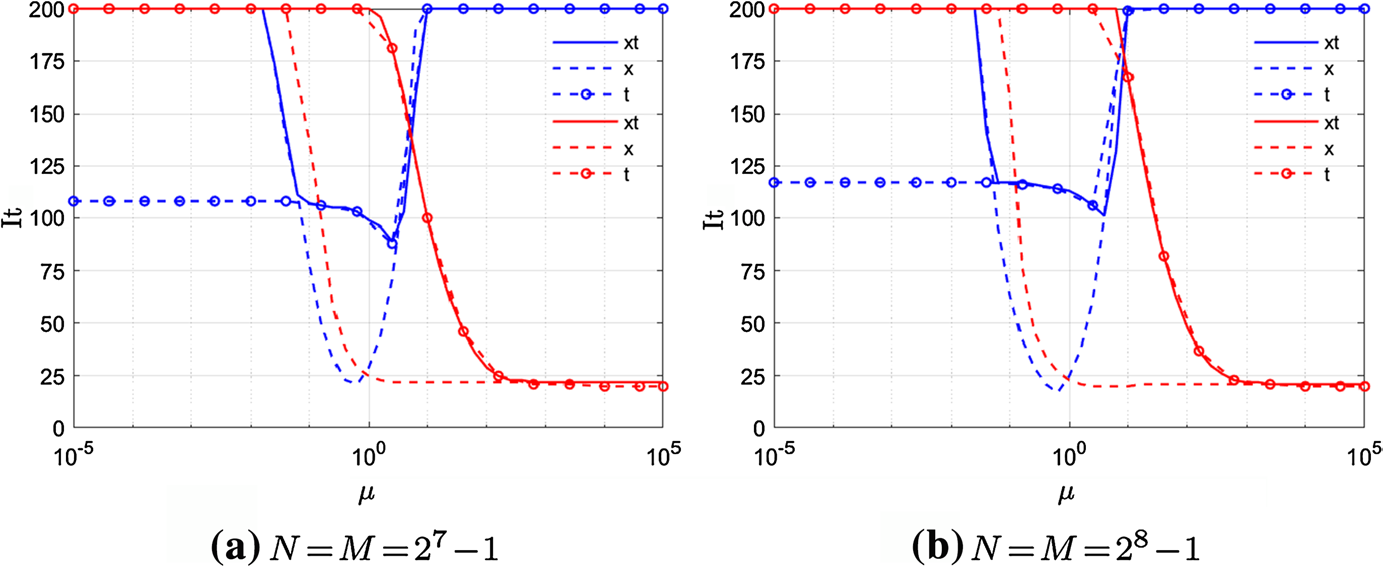
Example 2—V-cycle performances either using CN scheme (blue) or BDF2 scheme (red), and fixed $\alpha =1.5$

In Fig. 8, like in Sect. 6.4, we test the behavior of Vx, Vt, Vxt for two fine grids with $N=M=2^7-1$ and $N=M=2^8-1$.

We note that, as in the case of constant and equal diffusion coefficients, the results are not significantly sensitive to *N*, *M*. Moreover, like in Example 1, Vx is the only V-cycle, between the three tested, that yields good convergence results for both CN and BDF2. Finally, also in this variable coefficients example, the optimal convergence region, given by the magnitude of $\mu =d\cdot r$, is much bigger in the case of BDF2 than of CN.

We note that, independently of the constant or variable diffusion coefficients character, none of the tested methods is robust enough to deal with the case where $\mu <1$. Further tests, not reported here, show that this holds unchanged even when using the V-cycle as preconditioner for the GMRES. On the other hand, we stress that, since *d* is fixed, the choice of an opportune grid could lead to $\mu >1$, choosing $\varDelta t$ and $\varDelta x$ such that $d\varDelta t>2\varDelta x^\alpha$ and making V-cycle a suitable solver again.

### Two dimensional case

We end the numerical section by providing numerical results in the two-dimensional case. We consider the following extension of the one-dimensional FDE in Eq. (1):

10 $$\begin{aligned} \left\{ \begin{aligned} \frac{\partial u(x,y,t)}{\partial t}=&\ d_+ \frac{\partial ^{\alpha }u(x,y,t)}{\partial _{+}x^\alpha }+d_-\frac{\partial ^{\alpha }u(x,y,t)}{\partial _{-}x^\alpha }\\&\ e_+ \frac{\partial ^{\beta }u(x,y,t)}{\partial _{+}y^\beta }+e_-\frac{\partial ^{\beta }u(x,y,t)}{\partial _{-}y^\beta }+v(x,y,t),\\&\ \ \qquad \qquad \qquad \qquad \qquad \qquad \ \ \ (x,y,t)\in \varOmega \times [0,T],\\ u(x,y,t)=&0, \qquad \qquad \qquad \qquad \qquad \qquad \ (x,y,t)\in \left( \mathbb {R}^2\setminus \varOmega \right) \times [0,T],\\ u(x,y,0)=&u_0(x,y), \quad \qquad \qquad \qquad \qquad \quad \ \ (x,y)\in \overline{\varOmega }, \end{aligned} \right. \end{aligned}$$

where $\varOmega =(a_1,b_1)\times (a_2,b_2)$ is the space domain and $d_\pm ,e_\pm >0$ are the diffusion coefficients.

The discretization follows from the one-dimensional case and yields the same coefficient matrices as in Eq. (6), but with the extension through Kronecker product in two dimensions of each block. In the case where $d_+=d_-=e_+=e_-=d$, $\alpha =\beta$ and both spatial steps $\varDelta x,\varDelta y$ are equal, it holds that the grid dependent scale factor which multiplies the matrix representing the discretization in space is the same as in the one-dimensional case, i.e. $\mu =d\frac{\varDelta t}{2\varDelta x^\alpha }$.

#### Example 3

For our test we extend *Example* 1 to the two-dimensional case by assuming $d_\pm =e_\pm =d$, $\varOmega =(0,2)\times (0,2)$, and taking as final time step $T=1$. The true solution and the solution at the initial time $t=0$ are, respectively, given by

$$\begin{aligned} u_{ex}(x,y,t)=4\mathrm {e}^{-t} x^2(2-x)^2y^2(2-y)^2, \qquad u_0(x,y)=4x^2(2-x)^2y^2(2-y)^2. \end{aligned}$$

As done in *Example* 1, the numerical approximation of *v* is computed starting from the discretized exact solution.

Since the time-coarsening does not seems to be effective in the one-dimensional case, here we only consider the coarsening in both spatial dimensions and we use 0.95-BJ as post-smoother (higher weights seemed more suitable for this case).

Due to hardware limitations we cannot choose too dense grids, therefore we fix $N_x=N_y=M=2^6-1$, where $N_x$ and $N_y$ are the amount of points over the grids in the first and second spatial dimensions and *M* are the points over the time grid. Moreover, when considering the BDF2 scheme, as in the one-dimensional case we use CN to compute the solution at the first time step. As done in [11], we consider the case where the computation of the solution $u^{1}$ at the first the step is included in the coefficient matrix (inner CN) and we compare it with the previously considered case (outer CN), where $u^{1}$ is computed outside the coefficient matrix.

**Fig. 9:**
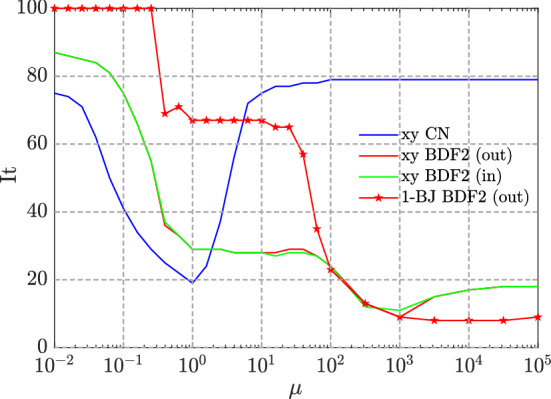
Example 3—Performances of 1-BJ using BDF2 with outer CN (starred line) and of V-cycle using CN scheme (blue), BDF2 scheme with outer CN (red), BDF2 scheme with inner CN (green) and fixed $\alpha =1.5$

In Fig. 9 we show the iterations to tolerance of the multigrid used as standalone solver (denoted by ‘xy’) for solving Eq. (10) discretized with CN, BDF2 with inner CN and BDF2 with outer CN. In the case of BDF2 with outer CN, we compare the results with 1-BJ.

We note that, even in the two-dimensional case, multigrid applied to CN is efficient when $\mu \approx 1$ and applied to BDF2 when $\mu \ge 1$. Moreover, we observe that the plots of BDF2 with inner and outer CN overlap almost everywhere, therefore the addition of CN inside the coefficient matrix does not seem to compromise the convergence of multigrid.

When $\mu \ge 10^2$, as in the one-dimensional case, 1-BJ is an efficient solver, since the coefficient matrix becomes block diagonally dominant.

## Conclusions and future works

In this work we focused on an all-at-once rephrasing of a time-dependent one-dimensional space-FDE with constant diffusion coefficients discretized with WSGD in space and CN or BDF2 in time. The unconditional stability of the BDF2-WSGD scheme has been proven, and the two-level Toeplitz structure of the resulting linear systems has been leveraged to design multigrid strategies that use block Jacobi as smoother and whose projectors definition is driven by the symbol.

We have numerically shown that V-cycle with semi-coarsening in *x* is the only multigrid, among all the tested ones, that yields good convergence results for both BDF2 and CN schemes. Moreover, it performs satisfactorily under less restrictive assumptions on the magnitude of $\mu =d\cdot r$ in the case of BDF2 than in the case of CN, and this let us to conclude that BDF2 is a much better alternative to CN for parallel-in-time integration with multigrid, when $\mu$ is large.

As future works, we plan to properly study the relaxation parameter of block Jacobi, as well as to consider alternative smoothers that could allow multigrid convergence even when $\mu \ll 1$. Moreover, it would be interesting to investigate the stability of higher order BDF schemes for smooth solutions in time, which could potentially increase the effectiveness of a multigrid solver reducing, at the same time, the overall computational cost. Furthermore, we aim at providing a parallel implementation of such strategies and to extend our structure-based approach also to other state-of-the-art solvers like parareal [25] or MGRIT [26]. Finally, we aim at extending our results also to the variable coefficients case and to higher-dimensional problems. This opens to other kind of anisotropies for which the V-cycle with semi-coarsening could not work anymore and that could, instead, be treated with the MG-S already applied to two-dimensional FDEs in [5].
